# Pneumothorax associated with nontuberculous mycobacteria: A retrospective study of 69 patients: Erratum

**DOI:** 10.1097/01.md.0000503498.80336.46

**Published:** 2016-10-07

In the article “Pneumothorax associated with nontuberculous mycobacteria: A retrospective study of 69 patients”,^[1]^ which appeared in Volume 95, Issue 29 of *Medicine*, Dr. Ueyama's first name was originally omitted. The article has since been corrected online.
